# The Effect of Chief Executive Officer and Board Prior Corporate Social Responsibility Experiences on Their Focal Firm’s Corporate Social Responsibility: The Moderating Effect of Chief Executive Officer Overconfidence

**DOI:** 10.3389/fpsyg.2022.891331

**Published:** 2022-07-22

**Authors:** Marwan Al-Shammari, Hussam Al-Shammari, Soumendra Nath Banerjee, D. Harold Doty

**Affiliations:** ^1^Soules College of Business, The University of Texas at Tyler, Tyler, TX, United States; ^2^Eberly College of Business, Indiana University of Pennsylvania, Indiana, PA, United States; ^3^College of Business, Misericordia University, Dallas, PA, United States

**Keywords:** CEO prior CSR, board prior CSR, CEO overconfidence, CEO interests, firm’s CSR

## Abstract

This research aims to examine how the prior experiences of the chief executive officer (CEO) and board influence the focal firm’s Corporate Social Responsibility (CSR) activities. Further, the present study examines how CEO overconfidence influences the diffusion of CSR activities. The authors theorize that overconfident CEOs are influenced more by the corporate strategies they experienced on other boards and less by the corporate strategies experienced by other directors. Through longitudinal analyses of the CSR profiles a sample of S&P 500 companies for the period 2006-2013, the study shows that CEO and board prior CSR experience are positively related to the firm’s current CSR activities. The authors find a significant positive moderating effect of CEO overconfidence on the relationship between CEO prior CSR and the focal firm’s CSR. The theory and results highlight how CEO and board prior CSR exposure may influence the focal firm’s stances toward CSR and that CEO overconfidence may have differential effects on these relationships.

## Introduction

One outcome of the increasing levels of social awareness and domestic activism in the United States and worldwide is that corporations are becoming increasingly aware of and concerned about their CSR image (Rodriguez-Fernandez, 2016; Banerjee, 2018). Perhaps as a result of previous public relations failures and the mistakes of a previous generation of managers, current managers seem more motivated than ever to use public CSR activities to improve the public’s perceptions about corporate managers (Schrempf-Stirling et al., 2016). As part of that effort, corporate managers are allocating more resources to CSR activities and being more deliberate when communicating their CSR commitment to stockholders, stakeholders, and the public at large.

Greater CSR focus within corporations has been paralleled by correspondingly greater prominence of CSR in the academic literature (Hoffmann, 2018). Not surprisingly, much of the initial focus of CSR research debated the effects on CSR activities on the firm’s financial performance. Despite the large number of studies on the relationship between CSR and firm performance, recent reviews have concluded that the exact nature of this relationship remains ambiguous and inconclusive (McWilliams and Siegel, 2000; Gras-Gil et al., 2016; Mellahi et al., 2016; Wang et al., 2016).

A second key focus in the literature has been on the antecedents of CSR initiatives. The examination of CSR antecedents has focused mostly on external factors that motivate managers and their organizations to engage in certain activities that are meant as responses to the demands of multiple stakeholders (Yang and Rivers, 2009; Othman et al., 2011; Lee et al., 2018; Scheidler et al., 2019). Considerable research has focused on stakeholder pressures and activism as major forces driving firms’ increasing attention to CSR (Doh and Guay, 2006; Wolf, 2014; Uldam and Hansen, 2017). Other studies have examined external factors such as the effects of legal mandates (Foote, 1984; Amor-Esteban et al., 2018), institutional pressure (Yang and Rivers, 2009; Young and Makhija, 2014), and industry and competition (Kim et al., 2018; Gras and Krause, 2020).

More recently, the focus on CSR antecedents has begun to shift from contextual variables to within firm variables (Chin et al., 2013; Cook and Glass, 2018). This stream of inquiry builds heavily on (Hambrick and Mason, 1984) upper echelons theory which posits that attributes of the top management team, and the CEO in particular, are key drivers of the firm’s decision process. More specifically, Hambrick and Mason (1984) contended that the personality of the top executives, the implicit knowledge that they have accumulated over years of experiences, and their prior experiences impact the way they perceive and process information and, ultimately, the decisions they make. Despite the attention that upper echelons theory has received, surprisingly little attention has been paid either to the CEO’s or to the board’s prior CSR experience. CEOs as well as the board members are key decision makers in organizations, and their prior experiences can significantly influence their decisions and shape their actions (Weng and Lin, 2014; Zhu and Westphal, 2014; Hamori and Koyuncu, 2015; Le and Kroll, 2017). Thus, a primary objective of the current effort is to focus directly on how prior CSR experience influences the upper echelon’s current CSR strategies.

More attention has been focused on the personality traits of chief executive officers. Of the many personality dimensions, CEO overconfidence has drawn increasing attention from strategy researchers in recent years (Schumacher et al., 2020). Overconfidence is typically associated with single-mindedness, entitlement, and a sense of superiority (Engelen et al., 2015; Bi et al., 2016; Tenney et al., 2019). Overconfidence is also associated with three different behavioral manifestations: overprecision which refers to inflated confidence in the accuracy of one’s beliefs, overplacement which refers to an inflated perception of one’s individual characteristics relative to others, and overestimation which refers to an inflated view of one’s ability, performance, success, and/or control of events (Moore and Healy, 2008; Macenczak et al., 2016; Schumacher et al., 2020). The psychology literature provides an abundance of evidence that individuals tend to overestimate their personal abilities. provides an abundance of that -on average- people overvalue their own abilities (Galasso and Simcoe, 2011; Aabo and Eriksen, 2018).

Chief executive officer’s may be more inclined to suffer from overconfidence bias than others because this problem is more common among talented individuals (Moore and Cain, 2007; Hogarth and Karelaia, 2012). Overconfidence affects the way that business leaders perceive phenomena, treat people, interpret information, and act upon these interpretations (Galasso and Simcoe, 2011; Chen et al., 2015). The more overconfident the CEO, the more likely the CEO will be to value their prior experiences and commit to their own beliefs and ignore or override the beliefs of others who might hold different beliefs (Zhu and Chen, 2015; Schoenherr et al., 2018). In light of these potential effects, a second objective of our study is to examine how CEO overconfidence effects the relationships between past CSR experiences of both the CEO and board members and current CSR policies of the focal firm.

Our study contributes to both the upper echelons theory and the research on CSR in several ways. First, we advance upper echelons theory by moving from coarse-grained demographic proxies to more fine-grained aspects of executive experience by specifically examining prior CSR experience and exposure of the key decision makers, both the CEO and members of the board of directors. Second, we contribute to CSR research by examining how CEO overconfidence, a salient internal driver, may affect how CEOs might overvalue/undervalue their own experiences and those of the board members. In doing so, we advance the literature on the factors that affect the extent to which CEOs might engage in CSR in their focal firms using their prior CSR experiences as an important driver. Additionally, we examine CEO overconfidence as an important contingency that may influence the extent to which CEO prior experiences may affect the focal firm’s CSR. Lastly, we also examine the effects of prior experiences of the board and the extent to which it might be influenced by the CEO overconfidence.

## Literature Review and Hypotheses Development

There is considerable evidence that top managers’, and especially CEOs’, characteristics have significant effects on the decision-making processes inside organizations, and thus on the organization’s strategies and outcomes (Hambrick, 2007; Quigley and Hambrick, 2015; Petrenko et al., 2016). Key CEO characteristics include age (Zhang et al., 2016; Belenzon et al., 2019), tenure (Hambrick and Fukutomi, 1991; Boling et al., 2016; Oh et al., 2018), educational background (King et al., 2016; Wang and Yin, 2018), and functional background (Koyuncu et al., 2010; Buyl et al., 2011). Such characteristics are influential in determining key strategic outcomes and policies including R&D spending (Barker and Mueller, 2002; Gwynne, 2003), voluntary environmental information disclosure (Lewis et al., 2014), risk taking and entrepreneurial orientation (Cao et al., 2015; Oesterle et al., 2016), and organizational culture (Giberson et al., 2009).

These individual characteristics are important because the strategic decision-making process is complex and ambiguous. TMT perceptions and interpretations become critical in this process (Dutton and Duncan, 1987a,b). Executives use their personalized interpretations of the circumstances they encounter, and these personalized interpretations are a function of the executives’ prior experiences, beliefs, values, and personality traits (Hambrick, 2007). We investigate the how the interplay between executive experience and executive personality influence CSR strategy. Specifically, we examine the moderating effect of CEO overconfidence on the relationship between CEO prior CSR exposure and the focal firm’s level of CSR, and the moderating effect of CEO overconfidence on the relationship between board prior CSR experience and the focal firm’s level of CSR.

### Prior Experience and Corporate Social Responsibility

Executives develop personal views and beliefs across their careers through accumulated experiences (Carpenter et al., 2004). The accumulation of prior experiences results in the development of what (Hambrick and Fukutomi, 1991) labeled the CEO paradigm. A key outcome of this paradigm is that the CEO’s beliefs and assumptions serve as “perceptual and interpretive apparatuses” for seeing a firm and its environment (Hambrick and Fukutomi, 1991: p. 721). This paradigm evolves over time and becomes the CEO’s knowledge base upon which they develop their beliefs and make decisions (Hambrick and Fukutomi, 1991). Thus, executives’ experiences and personalities impact subsequent strategic decisions and actions (Hambrick and Mason, 1984; Hambrick, 2007; Bailey, 2009).

By extension, we argue that a parallel set of processes are common across all upper-level executives including, in the current context, members of the Board of Directors. Board members also develop an appreciation for prior roles and experiences at other firms and are influenced by such experiences when participating in decisions at their current firms (Westphal and Milton, 2000; Whitler et al., 2018). Thus, like CEOs, Directors’ decisions about actions, decisions, and initiatives when responding to stakeholders’ demands are tied to beliefs that were developed from prior experiences (Westphal and Fredrickson, 2001).

Prior research on strategic decision making confirms that strategic choices are greatly influenced by managers’ personal backgrounds and prior experiences (Boeker, 1997b; Geletkanycz and Hambrick, 1997; Westphal and Milton, 2000; Zhu and Westphal, 2014) and that these prior experiences and exposures include serving as CEOs or board members (Zhu and Chen, 2015). The influence of prior experiences is particularly salient when executives face decision contexts similar to those they faced in previous firms (Useem, 1979; Haunschild, 1993; Westphal et al., 2001). Drawing on prior experiences enables executives and their organizations to gain legitimacy and reduce search and experimentation costs (DiMaggio and Powell, 1983).

There are numerous factors that contribute to the importance that executive experience plays in executive decisions. Executive migration between firms is one of the primary mechanisms through which organizational change occurs (Boeker, 1997a). When top managers move between organizations, they are likely to fall back on information and insights gained through their prior experience when making strategic decisions at the focal firm. In fact, CEOs with prior experiences are often hired to replicate their success in prior positions. Such CEOs are likely to have greater freedom and face less resistance when implementing strategies that are influenced by their past experiences (Hamori and Koyuncu, 2015). These successful executives are more likely than others to view themselves as superior, right, and more intelligent, and therefore are more likely to draw on their previously developed paradigms or knowledge base when making organizational decisions (John and Robins, 1994; Reina et al., 2014).

An executive’s prior experiences also facilitate information processing and generating alternatives for formulating strategic plans for the focal firm (Weng and Lin, 2014). The availability of relevant prior knowledge, exposure, and experience makes it more likely that decisions concerning certain situations will be biased and influenced by the relevant accumulated knowledge from prior experiences (Tversky and Kahneman, 1973) because managers develop scripts and schema over time. These scripts are influenced by prior experience in similar circumstances, increasing the likelihood of adopting similar strategies in the future (Hambrick and Mason, 1984; Hambrick et al., 1993; Westphal and Fredrickson, 2001).

Finally, when executives encounter highly uncertain decision settings, they typically have one of two responses. They may imitate what others firms are doing (DiMaggio and Powell, 1983) or, more likely, they will draw on experience and repeat what they did when faced with similar situations in the past. Decisions on CSR involve considerable uncertainty. Managers are often uncertain about the types of CSR activities to engage in and the amount of resources to devote to each activity. Additional uncertainty surrounds the performance outcomes and stakeholder reactions to various CSR activities (McWilliams and Siegel, 2000; Lepoutre et al., 2007). Given the uncertainties relating to activities and outcomes, it is only to be expected that CEOs and board members will draw from their scripts and prior experiences when considering strategic decisions (Zhu and Chen, 2015). For example, directors who have been part of a previous firm’s decision to dedicate more resources to engage in CSR activities such as charitable contributions, building facilities for local communities, and promoting environment-friendly policies will be favorably disposed toward such activities as strategies to achieve greater access to external resources, better image, and improved public relations. Therefore, they will be more likely to lean toward using the same strategies at their current firm because they are familiar and comfortable with such practices. Given that CEOs and Directors develop preferences toward strategies that they have experienced at other firms, CSR exposure at other firms is highly likely to be a primary factor that affects the focal firm’s CSR commitment. Therefore, we hypothesize:

**Hypothesis 1**. *Prior CSR experiences of the CEO at other firms will be positively related to the focal firm’s CSR.*

**Hypothesis 2**. *Prior CSR experiences of the board members at other firms will be positively related to the focal firm’s CSR.*

### The Moderating Effect of Chief Executive Officer Overconfidence

Overconfidence is one of the most studied CEO personality traits (Malmendier et al., 2011; Chen et al., 2015; Engelen et al., 2015; Aghazadeh et al., 2018). Prior research has documented that overconfident CEOs exhibit demonstrably different behaviors relative to other CEOs. For example, overconfident CEOs have stronger tendencies toward bold actions and risky strategies (Goel and Thakor, 2008; Bi et al., 2016; Aghazadeh et al., 2018), because they believe that they have more skills and a superior knowledge and experience compared to other CEOs. Overconfident CEOs believe that they have a superior ability to perform better and above average when making their investment policies, often leading to overinvestment (Ho et al., 2016). These behavioral differences have performance implication; firms run by overconfident CEOs have extreme and unstable financial performance (Andriosopoulos et al., 2013).

Because of their firm beliefs in their abilities and knowledge, as well as their commitment to optimism about their prior experiences, we argue that overconfident CEOs are more likely to be influenced by their prior experiences at other firms (either as CEO or as a board member). We contend that the motivational and cognitive elements of excessive confidence will play a role in explaining the degree to which overconfident CEOs will be influenced by prior experiences (Chatterjee and Hambrick, 2007, 2011; Zhu and Chen, 2015). The motivational aspect of excessive confidence suggests that such CEOs will interpret their prior behavior and actions more positively (Farwell and Wohlwend-Lloyd, 1998; Heimeriks, 2010) and even more positively when their behavior is highly publicly observable (Wallace and Baumeister, 2002a,b; Gerstner et al., 2013). Strategic initiatives and actions of firms are attributed to the CEO by the public and therefore such attention makes the CEO the public face of the firm. Similarly, it is unlikely that an overconfident CEO will interpret his or her prior behavior at other firms in a negative light. Such an interpretation would conflict with the current firm’s confidence in the CEO and would be inconsistent with the CEO’s self-esteem, self-admiration and it also (Campbell et al., 2004). These arguments are supported by the literature which suggests that overconfident CEOs are more likely than others to believe in the appropriateness of their prior behaviors, actions, and strategies (Heimeriks, 2010; Zhu and Chen, 2015).

The cognitive aspect of overconfidence suggests that overconfident CEOs believe that they have superior skills and abilities and a strong belief in their intelligence and proficiencies (John and Robins, 1994; Farwell and Wohlwend-Lloyd, 1998; Campbell et al., 2004; Wales et al., 2013). Their pursuit of prestige and uniqueness requires that they maintain such confidence and self-admiration (Chatterjee and Hambrick, 2011; Wales et al., 2013). They believe that they learn better than others (Moore and Cain, 2007; Heimeriks, 2010; Chen et al., 2015), and have stronger entitlement to their personal views (Paulhus and Williams, 2002; Campbell et al., 2004). They are likely to be confident about their superior interpretation and understanding of strategic actions based on their prior exposure to such actions in other firms because they are more likely to feel superior to others (Doukas and Petmezas, 2007; O’Reilly and Hall, 2021). When overconfident CEOs have been exposed to strong CSR activities and strong commitment toward societal and environmental concerns, they are confident about how to successfully engage in similar initiatives and strategies in their current firms. Therefore, both the motivational and cognitive elements of overconfidence suggest that the inclination of the CEO to pursue similar levels of CSR activities experienced in their previous firms is likely to be even stronger when the CEO is overconfident.

**Hypothesis 3**: *CEO overconfidence will positively moderate the relationship between the CEO’s prior CSR experiences at other firms and the focal firm’s overall CSR.*

Prior research suggests that a firm’s strategies are generally influenced by the prior experiences of top executives and directors at other firms and that these experiences result in the development of particular interpretations regarding specific corporate strategies (Chatterjee and Hambrick, 2007, 2011; Wales et al., 2013). The more overconfident an individual is, the less likely he or she is to accept criticism, opinions of others, or be influenced by others (Moore and Healy, 2008; Chu, 2012). Therefore, the more overconfident the CEO, the less likely the CEO will be influenced by other board members’ prior experiences (Zhu and Chen, 2015). People with excessive confidence are likely to dominate the decision-making process because of their need to assert their superiority and because of their overconfidence in their intelligence capabilities. In work settings such individuals tend to neglect other team members’ expertise in the decision-making processes (Campbell and Campbell, 2009; Campbell et al., 2011). Thus, extremely confident CEOs will project and assert their views, opinions, and beliefs through their interactions with other top management members, including members of the board. Further, the more overconfident the CEO is, the less likely that he or she will see as valuable other directors’ different experiences relative to a decision about any corporate strategy (Rhodes and Wood, 1992). Therefore, we propose overconfident CEOs will ignore the views of directors who are not in support of CSR and favor those members with positive prior CSR experiences.

**Hypothesis 4**: *CEO overconfidence will negatively moderate the relationship between board prior CSR experiences and the focal firm’s overall CSR.*

## Methodology

### Sample of Study

The initial sample for this study was the S&P 500 for the period 2006-2013. This includes both manufacturing and service firms. The use of S&P 500 firms as the sample for the study facilitates tracking the records of the directors’ prior appointments at other firms. From the original list of 500 firms, we identified firms in which the same CEO had remained in office during the entire period of the study.

Data relating to firms’ CSR activities were collected from the KLD database. In recent years, KLD has been the primary source of data for research on CSR activities of publicly traded firms because it is available for an extended period of time on a consistent basis. The results reported by the KLD specialists contain strengths and concerns in seven subject areas: human rights, corporate governance, diversity, employee relations, the environment, product characteristics, and community relations. The fact that these firms are rated by independent analysts adds to the credibility and the quality of the data (Hillman and Keim, 2001; Hong and Andersen, 2011).

Firms for which full CSR data was not available for the entire period were excluded from the study. We paid particular attention to the year in which the CEO became the chief executive officer and the year the members of the board had joined the firm to calculate the score of their prior CSR exposure. We excluded firms for which the CEO overconfidence measurement had incomplete or unavailable data. Our final sample consisted of 240 firms for the period 2006-2013, yielding 1338 observations.

We used Mergent-online database to collect data regarding CEO profiles and firm’s annual reports. We collected compensation data came from Execucomp. Press and media reports were collected from Factiva. Financial data were obtained from Compustat.

### Measures

#### Dependent Variable: Overall Corporate Social Responsibility

The KLD data comprises of two indices, one for the company’s strengths and the other for the company’s concerns. We operationalized the overall CSR score of the focal firm as the sum of strengths in the following dimensions (employee relations, community relations, environment, diversity, governance, and product quality) minus the sum of all concerns in these dimensions for each year. This is consistent with the approach followed in a number of prior studies (Graves and Waddock, 1994; Waddock and Graves, 1997; Wang and Choi, 2013; Petrenko et al., 2016).

#### Independent Variables: Chief Executive Officer Prior Corporate Social Responsibility Experience and Board Member Prior Corporate Social Responsibility Experience

Zhu and Chen (2015) measured CEO prior mergers and acquisitions experience by obtaining the level of mergers and acquisitions emphasis in their most recent firms before joining the focal firm. We used a similar approach to measure CEO prior CSR experience and the board’s prior CSR experience. We operationalized CEO prior experience by computing the average CSR score of each firm where the CEO had served in the three years prior to assuming the CEO position at the focal firm. We employed the same metric for individual board members, but then computed the mean within each firm’s board to get a single measure of board experience for each firm. Consistent with previous research, we weighted prior decisions by multiplying by 1, 2/3, and 1/3 for the year’s t-1, t-2, and t-3, respectively, to account for recency (e.g., Geletkanycz and Hambrick, 1997; Zhu and Chen, 2015) because recent experiences may have larger influences on subsequent decisions.

#### Moderator: Chief Executive Officer Overconfidence

Chief executive officer (CEO) overconfidence was measured based on how a CEO exercises stock options. Data were collected from the Execucomp database. Prior research on CEO overconfidence suggests that a CEO who retains more unexercised exercisable options is more confident about the future of the firm (Malmendier and Tate, 2005, 2008). Following previous studies (Dezsö and Ross, 2012; Lee et al., 2017), we first divided the annual value of the CEO’s holdings of vested, in-the-money options by the CEO’s total salary and bonus. We then applied a natural logarithmic transformation to the result to normalize the distribution of the measure.

#### Control Variables

We controlled for CEO tenure, natural logarithm of number of years as the CEO at the current firm, and CEO age, natural logarithm of a CEO’s biological age. We also controlled for several firm-level factors to increase the rigor of our findings. We controlled for firm financial performance operationalized as natural logarithm of Tobin’s Q. We controlled for a company’s age as the years since the firm first appeared in the Compustat data. We controlled for firm size, measured as the natural log of the firm’s total assets. The firm leverage is the summation of long-term debt and debt in current liabilities scaled by total assets. We controlled for firm slack as it has a direct influence on CEO preferences of both market and non-market strategies (Seifert et al., 2004; Julian and Ofori-dankwa, 2013). Consistent with prior studies, we calculated firm slack as the cash and cash equivalents scaled by total assets (Vanacker et al., 2017). Capital intensity is calculated as capital expenditures divided by sales. R&D (advertisement) intensity is computed as R&D (advertisement) expenditures scaled by total assets. Missing information on R&D expenditures and advertisement expenditures is a well-known issue in Compustat. Hence, following existing literature (Edmans et al., 2013; Koh and Reeb, 2015), we treat missing R&D expenditures and advertisement expenditures as zero and include dummy variables that pick-up the value 1 if non-missing data and 0 otherwise. We included year dummies and *SIC* two-digit industry dummies in all our models.

### Model Specification and Estimation Method

A minimum employment of either pooled OLS, the fixed effects (FE), or the random effects (RE) to panel data is recommended (Al-Shammari M. et al., 2021). The F-test null is strongly rejected in our present unbalanced sample, making FE an obvious choice over pooled OLS. Also, Hausman test null is strongly rejected, indicating superiority of FE even over RE. However, due to time-invariant nature of our independent variables, FE drops both variables. In addition, statistically significant results from Shapiro-Francia test on the dependent variable supports presence of non-normal distribution. Breusch-Pagan/Cook-Weisberg test further reveals the presence of heteroscedasticity. Additionally, Wooldridge test for autocorrelation proves presence of first-order autocorrelation. Hence, the feasible generalized least squares (FGLS) regression with heteroscedasticity and panel-specific AR1 autocorrelation is chosen as the appropriate means for analyzing the aforementioned hypotheses (Al-Shammari et al., 2022b).

In particular, we estimate the following model to predict CSR as a function of the explanatory variables discussed in the previous section.

$$C\,S\,R_{i\,t+1}=\alpha +\beta_{1}\,C\,E\,O\,p\,r\,i\,o\,r\,C\,S\,R_{i\,t}+\beta_{2}\,B\,o\,a\,r\,d\,p\,r\,i\,o\,r\,C\,S\,R_{i\,t}+\beta_{3}\,C\,E\,O\,p\,r\,i\,o\,r\,C\,S\,R_{i\,t}\,X\,C\,E\,O\,o\,v\,e\,r\,c\,o\,n\,f\,i\,d\,e\,n\,c\,e_{i\,t}$$

$$+\beta_{4}\,B\,o\,a\,r\,d\,p\,r\,i\,o\,r\,C\,S\,R_{i\,t}\,X\,C\,E\,O\,o\,v\,e\,r\,c\,o\,n\,f\,i\,d\,e\,n\,c\,e_{i\,t}+\beta_{n}\,X_{i\,t}+\gamma_{1}\,I\,n\,d\,u\,s\,t\,r\,y\,d\,u\,m\,m\,i\,e\,s+\delta_{1}\,Y\,e\,a\,r\,d\,u\,m\,m\,i\,e\,s+\varepsilon_{i\,t+1}$$

which *i* and *t* stand for an individual firm and specific year, respectively, in. CSR is a firm’s social performance, X denotes all other explanatory variables, including lagged CSR, and ε indicates the error term. We set β_2_, β_3_ and β_4_as zero to test the first hypothesis predicting positive association between CEO prior CSR and firm CSR. Whereas, β_1_, β_3_ and β_4_ are set to zero to test the second hypothesis anticipating Board prior CSR’s positive influence on a firm’s CSR. Coefficients β_3_ and β_4_ are required to illustrate hypothesis 3 and hypothesis 4, respectively, involving the moderating role of CEO overconfidence.

In case of the independent variables, endogeneity issue is unlikely because prior experiences have nothing to do with a focal firm. In other words, previous firms are the sources of both prior experiences – these experiences have already been earned even before a CEO or board member joins a focal firm. However, the moderator variable, CEO overconfidence, may contribute to such issue. Therefore, both Durbin-Wu-Hausman test and Wooldridge’s robust score test were resorted to for ensuring that the observed relationship between CEO overconfidence, CEO prior CSR experience, board prior CSR experience and firm-level social performance was not due to unobserved factors (Al-Shammari M. A. et al., 2021). Respective industry average is used as an instrument (even for both independent variables), which predict the dependent variable significantly. In the second stage regression with CSR as the dependent variable, the null is not rejected in both tests, implying endogeneity is not an issue in this case for the sample. Besides, we collect our dependent and all right-hand-side variables in t + 1 and t, respectively, because the relationship between dependent and independent variables may work better with a temporal lag, and it provides an initial protection against the endogeneity issue (Al-Shammari et al., 2022a).

## Empirical Results

Table 1 presents descriptive statistics of the sample. A typical firm has positive means of all variables. Although, a firm shows comparatively low values of Tobin’s Q, leverage, slack, and all three intensities. An average board prior CSR is greater than an average CEO prior CSR. Missing information is prevalent in case of both R&D and advertisement expenses. Next, we form group of firms based upon their CEO prior CSR and board prior CSR values. We note that firms with positive values of either of the experiences yield better CSR_*t+*1_ on average in the bottom panel of Table 1. We also observe that the differences between the means are highly significant as represented by their associated t-statistics in parentheses. These are the preliminary evidences in support of our H1 and H2. Further, including lags by a year to this annual data, both an augmented Dickey-Fuller test with or without trend and Phillips-Perron test reject the null of unit root test at 0.1% statistical significance level when CSR_*t+*1_ is the dependent variable. The test results support stationarity or stability of the CSR_*t+*1_ variable.

**TABLE 1:** Descriptive statistics.

Variables	M	SD	Median	Min.	Max.
CSR_*t+*1_	1.56	4.45	1.00	–8.00	18.00
CEO prior CSR_*t*_	2.46	3.51	1.40	–4.50	15.20
Board prior CSR_*t*_	3.19	1.59	3.31	–1.50	7.80
CEO overconfidence_*t*_	1.60	1.95	1.78	–7.03	20.01
CEO tenure_*t*_	1.74	0.78	1.79	0.00	3.78
CEO age_*t*_	4.03	0.11	4.04	3.61	4.44
CSR_*t*_	1.04	4.35	0.00	–9.00	18.00
Tobin’s Q_*t*_	0.54	0.39	0.49	–0.21	2.32
Firm age_*t*_	3.48	0.60	3.66	1.39	4.14
Size_*t*_	9.32	1.15	9.16	6.19	13.59
Leverage_*t*_	0.24	0.16	0.21	0.00	1.03
Slack_*t*_	0.11	0.11	0.08	0.00	0.76
Capital intensity_*t*_	0.06	0.10	0.03	0.00	1.02
R&D intensity_*t*_	0.02	0.04	0.00	0.00	0.68
Advertisement intensity_*t*_	0.02	0.04	0.00	0.00	0.47
R&D missing_*t*_	0.69	0.46	1.00	0.00	1.00
Advertisement missing_*t*_	0.47	0.50	0.00	0.00	1.00
	M (CEO prior CSR_*t*_ ≤ 0)		M (CEO prior CSR_*t*_ > 0)		Difference (t-stat)
CSR_*t+*1_	−0.62		2.70		−3.32 (−13.84)
	M (Board prior CSR_*t*_ ≤ 0)		M (Board prior CSR_*t*_ > 0)		Difference (t-stat)
CSR_*t+*1_	−1.70		1.79		−3.49 (−7.28)

*N = 1,338.*

Table 2 contains the pairwise correlations among the primary variables of the study. Our first variable of interest which is CEO prior CSR experience is significantly and positively correlated with CSR. Board prior CSR is also significantly correlated with CSR. CEO overconfidence is significant and positively correlated with CSR as well. Given the patterns and magnitudes of these inter-correlations, we examined variance inflation factors (VIF) produced from the main effects of our models to assess the potential for multicollinearity issues. The VIF values ranged from 1.20 to 3.58, which is less than 10. The mean VIF value is 1.64 is mean VIF, which is less than 6. Based on the ranges and the mean VIF value, multicollinearity does not appear to be a significant problem in the current data.

**TABLE 2:** Pairwise correlation matrix.

Variables	1	2	3	4	5	6	7	8	9	10	11	12	13	14	15	16
CSR*_*t+*1_*																
CEO prior CSR*_*t*_*	0.37***															
Board prior CSR*_*t*_*	0.27***	0.25***														
CEO overconfidence*_*t*_*	0.11***	0.03	0.10***													
CEO tenure*_*t*_*	–0.02	0.02	−0.08***	0.22***												
CEO age*_*t*_*	0.01	−0.09***	–0.03	0.07**	0.41***											
CSR*_*t*_*	0.84***	0.40***	0.27***	0.11***	–0.02	–0.01										
Tobin’s Q*_*t*_*	0.20***	0.17***	0.14***	0.39***	–0.04	−0.09***	0.23***									
Firm age*_*t*_*	0.23***	0.16***	0.20***	–0.03	−0.09***	0.07***	0.21***	–0.03								
Size*_*t*_*	0.24***	0.14***	0.23***	0.09***	−0.06**	0.14***	0.20***	−0.10***	0.42***							
Leverage*_*t*_*	–0.04	–0.04	−0.15***	−0.09***	0.02	0.07***	−0.05*	−0.22***	0.04	0.05*						
Slack*_*t*_*	0.18***	0.16***	0.26***	0.17***	0.01	−0.08***	0.19***	0.37***	−0.12***	−0.09***	−0.35***					
Capital intensity*_*t*_*	0.00	–0.03	−0.21***	0.03	0.07**	0.07***	0.00	−0.09***	0.00	0.21***	0.03	−0.10***				
R&D intensity*_*t*_*	0.21***	0.24***	0.22***	0.16***	0.00	–0.02	0.20***	0.34***	0.00	0.08***	−0.13***	0.42***	–0.04			
Advertisement intensity*_*t*_*	0.14***	0.14***	0.17***	0.06	−0.08***	−0.17***	0.16***	0.28***	−0.08***	−0.16***	–0.03	0.14***	−0.08***	0.04		
R&D missing*_*t*_*	0.16***	0.13***	0.12***	0.11***	–0.02	0.00	0.16***	0.25***	0.08***	0.01	–0.03	0.08***	−0.22***	0.31***	0.07***	
Advertisement missing*_*t*_*	0.16***	0.10***	0.21***	0.02	−0.08***	−0.08***	0.17***	0.12***	–0.01	–0.04	0.11***	0.01	−0.15***	–0.03	0.46***	0.12***

*p < 0.10.

**p < 0.05.

***p < 0.01.

Table 3 shows the results of our FGLS analysis. In the base model, Model 1, we included all the control variables, including the moderator, CEO overconfidence. We then added CEO prior CSR experiences to create Model 2. Model 3 is built on Model 2 by adding board prior CSR experiences. To create Model 4, we added the interaction between CEO prior CSR experience and overconfidence. In Model 5 we added the interaction between board prior CSR experience and CEO overconfidence to the Model 4 variables. Consistent with prior studies (Zhu and Chen, 2015), we interpret our results for first two hypotheses and last two hypotheses based on the fully specified Model 3 and Model 5 where we included all primary and interaction variables, respectively.

**TABLE 3:** Effects of chief executive officer (CEO)/Board prior Corporate Social Responsibility (CSR) on CSR and moderating effects of overconfidence.

Variable	Model 1	Model 2	Model 3	Model 4	Model 5
CEO prior CSR*_*t*_*	_	0.05***	0.05***	0.00	0.01
		(0.02)	(0.02)	(0.02)	(0.02)
Board prior CSR*_*t*_*	_	_	0.05*	0.05**	0.05
			(0.03)	(0.03)	(0.03)
CEO prior CSR*_*t*_* X CEO	_	_	_	0.02***	0.02***
overconfidence				(0.01)	(0.01)
Board prior CSR*_*t*_* X CEO	_	_	_	_	0.01
overconfidence					(0.01)
CEO overconfidence*_*t*_*	0.05**	0.05**	0.04**	0.00	−0.02
	(0.02)	(0.02)	(0.02)	(0.03)	(0.04)
CEO tenure*_*t*_*	−0.09*	−0.10*	−0.08	−0.07	−0.08
	(0.05)	(0.05)	(0.05)	(0.05)	(0.05)
CEO age*_*t*_*	0.11	0.16	0.12	−0.01	−0.04
	(0.36)	(0.37)	(0.38)	(0.37)	(0.36)
CSR*_*t*_*	0.78***	0.76***	0.77***	0.77***	0.77***
	(0.01)	(0.02)	(0.02)	(0.02)	(0.02)
Tobin’s Q*_*t*_*	−0.04	−0.10	−0.13	−0.11	−0.12
	(0.14)	(0.14)	(0.14)	(0.14)	(0.14)
Firm age*_*t*_*	0.18***	0.13*	0.10	0.09	0.10
	(0.07)	(0.07)	(0.08)	(0.07)	(0.07)
Size*_*t*_*	0.29***	0.27***	0.27***	0.27***	0.27***
	(0.05)	(0.05)	(0.05)	(0.05)	(0.05)
Leverage*_*t*_*	−0.11	−0.10	−0.20	−0.23	−0.20
	(0.22)	(0.23)	(0.25)	(0.24)	(0.24)
Slack*_*t*_*	−0.29	−0.21	−0.34	−0.59	−0.60
	(0.45)	(0.50)	(0.52)	(0.52)	(0.52)
Capital intensity*_*t*_*	−0.55	−0.58	−0.37	−0.42	−0.39
	(0.45)	(0.46)	(0.49)	(0.48)	(0.49)
R&D intensity*_*t*_*	4.33***	4.28***	4.29***	4.54***	4.45***
	(1.08)	(1.33)	(1.34)	(1.44)	(1.44)
Advertisement intensity*_*t*_*	6.30***	5.89***	5.92***	6.00***	6.04***
	(1.97)	(1.98)	(2.02)	(2.07)	(2.07)
R&D missing*_*t*_*	0.28**	0.30***	0.28**	0.24**	0.23*
	(0.11)	(0.11)	(0.12)	(0.12)	(0.12)
Advertisement missing*_*t*_*	−0.05	−0.03	−0.03	−0.02	−0.02
	(0.13)	(0.13)	(0.13)	(0.13)	(0.14)
Intercep*_*t*_*	−3.95**	−3.65**	−3.41**	−2.85*	−2.67*
	(1.55)	(1.60)	(1.61)	(1.53)	(1.53)
N	1,338	1,338	1,338	1,338	1,338
Wald test (compared to)			14.16*** (1)	27.41*** (1)	28.31*** (1)

*Industry dummy variables and year dummy variables are included in all models. Standard errors are in parentheses.*

*p < 0.10.

**p < 0.05.

***p < 0.01.

Our first hypothesis predicts that higher a CEO’s prior CSR experience, higher the firm’s social performance. FGLS yields a positive and significant β_1_ (β = 0.05, ρ < 0.01) in Model 3. Hence, Model 3 does provide support to hypothesis 1 that suggested a positive relationship between CEO prior CSR experience and the focal firm’s CSR.

Again, FGLS yields a positive and significant β_2_ (β = 0.05, ρ < 0.10) in Model 3. This result strongly supports hypothesis 2 that predicts a positive impact on focal firm’s CSR of board prior CSR experience.

As per hypothesis 3, CEO overconfidence is supposed to positively moderate the relationship between CEO prior CSR and the focal firm’s CSR. FGLS produces positive and statistically significant β_3_ (β = 0.02, ρ < 0.01) in Model 5. Hence, the fact that CEO overconfidence strengthens the relationship between CEO prior CSR and a firm’s social performance is fully supported for the sample.

CEO overconfidence is also assumed to negatively moderate the relationship between board prior CSR experience and a firm’s social performance, according to hypothesis 4. The results, however, show a positive and not significant effect β_4_ (β = 0.01, ns) as shown in Model 5 Hence, hypothesis 4 is not supported.

**FIGURE 1:**
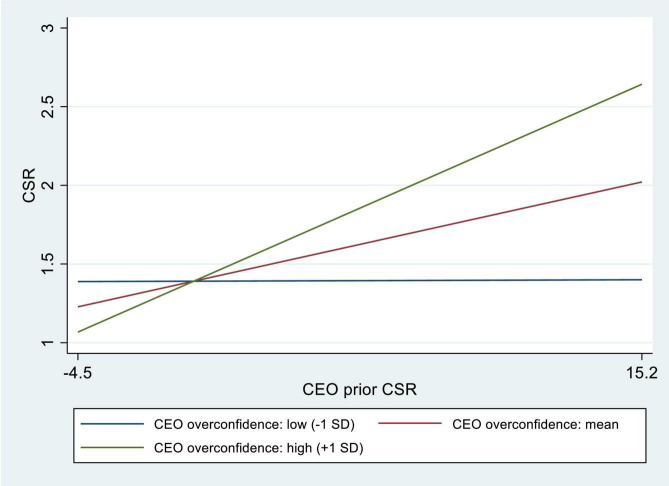
Moderating role of chief executive officer (CEO) overconfidence in the firm CSR-CEO Prior Corporate Social Responsibility (CSR) relationship.

In order to interpret the statistically significant interaction effect between CEO prior CSR experience and CEO overconfidence in predicting an average firm’s social performance, we graph it in Figure 1.

Figure 1 confirms that a firm with 1 sd above mean or mean CEO prior experience observes better immediate future social performance compared to its counterparts with 1 sd below mean CEO prior experience.

### Robustness Check

To ensure the robustness of our results, we reran the analysis using a substitute measure for social performance, namely z-score CSR and CSR strengths after controlling for CSR concerns. As additional robustness checks, we used different measures for firm size (natural log of firm sales and natural log of firm assets) and different measures for R&D and advertisement intensities (first, scaling by sale, and then, scaling by number of employees). We also ran the analysis by replacing *SIC* two-digit industry categories by Fama-French industry classification. These robustness tests had no effect on the substantive results; the first three hypotheses continue to be supported; the fourth hypothesis is not supported.

A CEO may not be able to practice her/his full power at the current office in the first year irrespective of prior CSR experience level. Hence, we rerun all regressions dropping CEOs with just one year of experience. First three hypotheses still hold strong.

Results related to both hypotheses involving CEO prior CSR experience remain unchanged even after rerunning all our regressions including industry level average variables like average industry Tobin’s Q, average industry size, average industry leverage, average industry slack, average industry capital intensity, average industry R&D and advertisement intensities.

To avoid any type of recessionary impact from the great financial crisis of 2007-2009, all regressions were rerun for the 2010-2013 period. First three hypotheses remained supported.

## Discussion

Drawing on both institutional theory (e.g., DiMaggio and Powell, 1983) and upper echelon theory (e.g., Hambrick and Mason, 1984) we proposed that both CEO and board prior CSR exposure would positively influence the focal firm’s CSR strategy. We also hypothesized that CEO overconfidence would have a positive moderating influence on these relationships. Our study found a positive and significant relationship between CEO prior CSR experience and the focal firm’s CSR. Additionally, we found that overconfidence positively affects this relationship. These results indicate that overconfident CEOs are more likely than others to embrace policies that are similar to the policies they have experienced at previous firms whether as board members or as CEOs. It adds to our understanding of the literature on policy migration and inter-organizational imitation.

Our results also indicate that prior CSR experience of the board has a positive impact on the firm’s CSR. Serving on the boards of other companies which have high levels of involvement in CSR activities results in those directors advancing the CSR agenda in the focal organization as well. This would suggest that corporate practices diffuse over time and that one of the mechanisms of such diffusion may be common membership by those in decision making roles. While we found no moderating effect of CEO overconfidence on the relationship between CEO prior CSR experience and CSR, we found a positive and non-significant moderating relationship between board prior experience and focal firm CSR. This is clearly an intriguing result. It is possible that highly overconfident CEOs maybe willing to be influenced by the board prior CSR experiences for two reasons. First, the credit for the firm’s CSR activities can be largely claimed by the CEO. Second, indulging the board’s preferences with regard to CSR may elicit greater support from the board for the CEO’s initiatives in areas such as M&As, thus facilitating the overconfident CEO’s risky actions. That is, overconfident CEOs might be drawn to bolder market-focused strategies such as disruptive innovation, mergers and acquisitions, international alliances, and other risky strategies, and therefore would need more resources to engage in such strategies. Such actions would need the support and the approval of the board. CEOs may be allowing the board members to influence the firm’s CSR strategy in exchange for support for the CEO’s strategic decisions in other areas.

Our study makes important contributions to several areas of the literature. First, we contribute to the literature on inter-organizational imitation (DiMaggio and Powell, 1983) by highlighting the impact of prior CSR experience on the CEO and board. In doing so, we take a modest step in addressing the imbalance in prior literature which has primarily focused on external determinants of CSR. One possible avenue for future research is the learning effects and its subsequent performance implications of prior CSR experience of the CEO and the board. That is, it is important to examine if CEO and board members with prior CSR experience create a stronger CSR-performance relationship than CEOs and board members who have less experience with CSR.

We also contribute to the growing literature on the impact of personality traits of organizational leaders on their CSR engagements (e.g., Chatterjee and Hambrick, 2011), namely CEO overconfidence. CEO overconfidence has been recognized as an increasingly important personality trait that influences a firm’s strategic orientation (Goel and Thakor, 2008; Andriosopoulos et al., 2013; Aghazadeh et al., 2018). It has also been found that most organizational leaders have some degree of overconfidence (Galasso and Simcoe, 2011; Chen et al., 2015). However, CEOs vary in the extent to which they possess this personality trait and this variance may have important implications for the strategies that they choose and the consequent resource commitments they make (Gerstner et al., 2013; Cragun et al., 2020). Yet, most studies have focused on the linkages between CEO overconfidence and market strategies.

Recently, CEO overconfidence has been found to have an equal impact on non-market strategies as well (Tang et al., 2015; McCarthy et al., 2017). Overconfidence has been found to be negatively related to CSR (McCarthy et al., 2017), whereas narcissism positively affected CSR and, even more interestingly, it has been found to negatively moderate the relationship between CSR and firm performance (Petrenko et al., 2016). Yet another potential avenue for future research is the examination of the persistence (or lack thereof) of specific strategies from prior experiences based on the personality traits of the CEO. That is, certain strategies may be less affected by personality traits than others. Future research could also examine the status of the prior firms on which the CEO and the board have previously served to see whether the status of those firms (highly prestigious, prominent, profitable) has a moderating effect on the extent to which the CEO and board experiences in those companies impacts their subsequent strategic orientations, priorities, and preferences.

Although there has been increasing research attention on how boards of directors may influence firms’ strategic decisions [see review by Westphal and Zajac (2013)], there has been limited awareness that the directors’ impact on strategy may be contingent on the personality traits of the CEO. While our finding that CEO overconfidence moderates the influence of other directors’ experience on major corporate decisions, namely CSR, was not significant and contrary to what we predicted, it would be worth examining whether other salient personality traits of the CEO may increase or decrease the influence and authority of the board in certain strategic contexts such as CSR. Further, CEO overconfidence may significantly affect decision processes and alter decision outcomes in other strategic contexts. The positive moderation effect of CEO overconfidence on the relationship between board prior CSR experiences and the firm’s CSR may imply a certain ingratiation behavior. Prior research suggests that overconfidence is almost always present in narcissistic individuals (McCarthy et al., 2017), and, given the positive coefficient of the CEO overconfidence in the case of board prior experiences, it may be the case that narcissistic CEOs may let the board initiate positive or negative CSR strategies consistent with the board prior CSR exposure and their CSR preferences in exchange for a greater freedom for the CEO to engage in other bolder actions that would not necessarily benefit shareholders. Zhu and Chen (2015) for example found that narcissistic CEOs are more drawn to bold actions such as mergers and acquisitions. By giving the board more authority in CSR decisions, CEOs may secure the board’s support to engage in other strategies such as M&As.

## Conclusion

Organization researchers have long been interested in explaining why organizations become similar to each other over time (DiMaggio and Powell, 1983; Zhu and Chen, 2015). One explanation for increasing isomorphism in organizational fields is that managers remain committed to certain paths and strategies they have experienced and applied throughout their careers (Westphal and Fredrickson, 2001; Yang et al., 2011; Zhu and Chen, 2015). CEOs and Directors have prior experiences as either CEOs, board members, or C-Suit members prior to their current positions at the focal firm. These experiences have been shown to influence their subsequent actions and decisions. Zhu and Chen (2015), for example, found that prior merger and acquisition experiences of CEOs and boards influence their current firm’s M&A strategies. We have extended the exploration of CEO and Board prior experience is to examine how prior experience impacts the performance effect of CSR. We would expect that when the board members have higher CSR experiences, they would be able to increase the consistency of the firm’s CSR strategies, balance the pace of CSR engagements, and align these strategies with the best interests of shareholders and other key stakeholders. The same logic may apply to the CEO and board’s prior CSR experiences over a broad range of domains such as innovation, international expansion, and perhaps even creative disruption strategies.

Our work also highlights the need for greater understanding of the micro foundations of executive behaviors and actions. To date inadequate attention has been focused on the possibility that CEO personality traits may have an effect on how prior experience impacts executives subsequent actions. Some personality traits may either amplify or suppress the effects executives prior experiences at other firms. In the present study, we examine the implications of CEO prior CSR experiences, board prior CSR experiences, and CEO overconfidence on the focal firm’s CSR. Specifically, we examine whether CEO/board prior CSR experiences will influence the focal firm’s CSR, and whether CEO overconfidence as a fundamental personality trait among organizational leaders will have a moderating effect on the relationships between CEO/Board prior CSR and the focal firm’s CSR. We suspect that CEO overconfidence is only one of many different personality characteristics that affect executive decision making. Thus we believe that the integration of personality theories with executive decision making behaviors will become another productive area for ongoing research.

### Limitations

As with all studies, interpreting our current work requires recognition of several potential limitations. For example, data availability problems restricted our sample to a small subset of Fortune 500 firms which restricts the generalizability of our results. All of the firms in our sample are large, publicly traded corporations. Additional studies will need to determine whether the current results generalize across smaller firms with different ownership structures. Our operationalization of CEO and board prior experience looked at only the most recent three years of executives’ experience. As a result, we can only speculate about the influence of early career experiences in shaping managerial preferences. More fine-grained information about the career paths of individual executives might shed light interesting developmental process and influence that have long lasting effects on an executives’ behavior and values in later stages if their careers. Our reliance on the KLD database as the data source for firm’s CSR activities, our results come with the inherent limitations of the KLH data. Future studies, therefore, could use more direct measures of CSR or complementary measures from other sources. Finally, the overconfidence measures we use is based on unobtrusive indicators due to the difficulty of obtaining CEO responses to surveys. Using richer and more direct measures of CEO overconfidence in future studies could help generate a more nuanced understanding of behavioral differences between CEOs who are overconfident versus those who are merely confident.

## Data Availability Statement

The original contributions presented in this study are included in the article/supplementary material, further inquiries can be directed to the corresponding author.

## Author Contributions

All authors listed have made a substantial, direct, and intellectual contribution to the work, and approved it for publication.

## Conflict of Interest

The authors declare that the research was conducted in the absence of any commercial or financial relationships that could be construed as a potential conflict of interest.

## Publisher’s Note

All claims expressed in this article are solely those of the authors and do not necessarily represent those of their affiliated organizations, or those of the publisher, the editors and the reviewers. Any product that may be evaluated in this article, or claim that may be made by its manufacturer, is not guaranteed or endorsed by the publisher.
